# Transient Receptor Potential Channel 6 Knockout Ameliorates Kidney Fibrosis by Inhibition of Epithelial–Mesenchymal Transition

**DOI:** 10.3389/fcell.2020.602703

**Published:** 2021-01-15

**Authors:** Yanhong Zhang, Nina Yin, Anbang Sun, Qifang Wu, Wenzhu Hu, Xin Hou, Xixi Zeng, Min Zhu, Yanhong Liao

**Affiliations:** ^1^Department of Anatomy, Tongji Medical College, Huazhong University of Science and Technology, Wuhan, China; ^2^Department of Anatomy, College of Basic Medicine, Hubei University of Chinese Medicine, Wuhan, China; ^3^Key Laboratory of Neurological Diseases of Ministry of Education, Tongji Medical College, Huazhong University of Science and Technology, Wuhan, China; ^4^Department of Thoracic Surgery, Tongji Hospital, Tongji Medical College, Huazhong University of Science and Technology, Wuhan, China

**Keywords:** EMT, TRPC6, TEC, UUO, kidney fibrosis, TGF-β1

## Abstract

Kidney fibrosis is generally confirmed to have a significant role in chronic kidney disease, resulting in end-stage kidney failure. Epithelial–mesenchymal transition (EMT) is an important molecular mechanism contributing to fibrosis. Tubular epithelial cells (TEC), the major component of kidney parenchyma, are vulnerable to different types of injuries and are a significant source of myofibroblast by EMT. Furthermore, TRPC6 knockout plays an anti-fibrotic role in ameliorating kidney damage. However, the relationship between TRPC6 and EMT is unknown. In this study, TRPC6^−/−^ and wild-type (WT) mice were subjected to a unilateral ureteric obstruction (UUO) operation. Primary TEC were treated with TGF-β1. Western blot and immunofluorescence data showed that fibrotic injuries alleviated with the inhibition of EMT in TRPC6^−/−^ mice compared to WT mice. The activation of AKT-mTOR and ERK1/2 pathways was down-regulated in the TRPC6^−/−^ mice, while the loss of Na^+^/K^+^-ATPase and APQ1 was partially recovered. We conclude that TRPC6 knockout may ameliorate kidney fibrosis by inhibition of EMT through down-regulating the AKT-mTOR and ERK1/2 pathways. This could contribute to the development of effective therapeutic strategies on chronic kidney diseases.

## Introduction

Kidney fibrosis is the end-stage of all progressive chronic kidney diseases, which can be caused by urinary obstruction and may develop into end-stage renal disease (Paniagua-Sierra and Galvan-Plata, 2017). Kidney fibrosis, including interstitial fibrosis, is characterized by proliferation of tubular epithelial cells (TEC) and interstitial cells, loss of glomerular and peritubular capillary architecture, and accumulation of the extracellular matrix (ECM) (Liu, 2011). Furthermore, the reduction of kidney parenchyma function is strongly related to the kidney fibrosis, which leads to compromised kidney insufficiency (Zeisberg and Kalluri, 2013). TEC, the major component of kidney parenchyma, often suffer ill-effects from different types of kidney injuries (Liu, 2004). As such, an increasing number of studies on kidney fibrosis have been devoted to the exploration of TEC. Previous studies have shown that exploring the complex cellular mediators and molecular mechanisms of kidney fibrosis could offer new treatment avenues for chronic kidney disease (Bechtel et al., 2010; Sugimoto et al., 2012; LeBleu et al., 2013). However, no effective treatment to inhibit the onset or progress of chronic kidney disease has been discovered, and the occurrence of kidney fibrosis is still on the rise. Therefore, further investigation in kidney fibrosis is urgently needed.

In recent years, kidney fibrosis studies have shown that TEC, rather than being victims or bystanders, are likely an originator of the interstitial fibrosis reaction to a variety of injuries (Gewin, 2018; Liu et al., 2018). Importantly, TEC, as a vital source of myofibroblasts, contribute to kidney fibrosis by transitioning cells to mesenchymal characteristics (Iwano et al., 2002; Kalluri and Weinberg, 2009). The process is known as epithelial–mesenchymal transition (EMT) and may be induced by TGF-β1 (Hay, 1995). Many studies have suggested that EMT is a significant process in kidney fibrosis (Lovisa et al., 2015, 2016). Generally speaking, during EMT, epithelial cells lose their junctions, break apical–basal polarity, remodel their cytoskeleton, and experience signal process changes that define cell shape and reset gene expression (Thiery and Sleeman, 2006; Thiery et al., 2009). Epithelial cells then reveal some plasticity and lose epithelial markers such as E-cadherin (E-cad) and Cadherin-16 (Cadh16). Cadh16, as a kidney-specific cadherin, is present in kidney tubular epithelial cells (Grande et al., 2015). Simultaneously, other changes occur, such as the development of spindle-shaped morphology, which is characteristic of mesenchymal cell lines, and the improved producing capacity of extracellular matrix. In addition, during EMT, cells express mesenchymal-related molecular markers, such as α-SMA and vimentin (Radisky et al., 2007; Quaggin and Kapus, 2011), and the transcription factor snail1 is activated, which inhibits the expression of epithelial genes (Barrallo-Gimeno and Nieto, 2005; Peinado et al., 2007; Xu et al., 2009), especially E-cad and Cadh16 (Horikawa et al., 2011; Lin X. et al., 2019).

It is well-known that EMT has been observed in tissue modeling and remodeling (Katz et al., 2002; Coresh et al., 2007) and is integral to embryonic development and the physiopathology mechanism reactivated in wound healing, fibrosis, and cancer progression (Chapman, 2011; Liu et al., 2014; Gewin, 2018). Of note, EMT is triggered by pleiotropic signaling factors, including the TGFβ superfamily, epidermal growth factor (EGF), Sonic Hedgehog (Shh), fibroblast growth factor (FGF), and Wnt/β-catenin. TGF-β1 is an especially powerful inducer of EMT *in vitro* and *in vivo* and has been shown to be important in the activation and maintenance of fibrosis (Tamaki et al., 1994; Eddy, 1996; Holian et al., 2008). Fibrosis were initiated by TGF-β1 through Smad-dependent (canonical) and Smad-independent (non-canonical) pathways. Several Smad-independent pathways were activated in the process of EMT including the phosphatidylinositol-3′-kinase (PI3K), AKT (Pillow et al., 2014), ERK1/2, p38, and Ras- and Rho-GTPases (Moustakas and Heldin, 2005; Zhang, 2018). Furthermore, the AKT-mTOR pathway is activated to produce the transcriptional factor snail1, which can suppress the expression of E-cad in the process of EMT (Grille et al., 2003). ERK1/2 pathway is also activated in EMT and induced by TGF-β1 (Grande et al., 2002; Xie et al., 2004). Appropriate EMT induced by injury is beneficial; however, exaggerated EMT caused by healing can lead to tissue scarring or fibrosis. In addition to findings in experimental animal models, EMT of TEC was also detected in human fibrotic kidneys and was associated with disease progression (Rastaldi, 2006; Hertig et al., 2008).

Transient receptor potential channels (TRPCs) were first observed in *Drosophila*, in which the photoreceptors exhibited a transient voltage response when treated with a continuous light as trp gene mutations equipped (Minke, 1977; Montell et al., 1985). Distinct from other ion channels, TRPCs are classified by their homology and not by the function of ligand or selectivity, as TRPCs are too complex to elucidate their function. TRPC1 forms heteromeric channels with TRPC4 and/or TRPC5 and was the first mammalian TRPC reported (Wes et al., 1995). TRPC3, TRPC6, and TRPC7 proteins share 75% identity and are sensitive to the intracellular concentration of Ca^2+^. TRPC2 seems to be a pseudogene in humans. TRPC6 is strongly involved in kidney diseases on the basis of wide expression in kidney cells, such as podocytes, glomerular mesangial cells, and TEC (Shen et al., 2013, 2014; Schlondorff, 2017; Wu et al., 2017). Mutation in TRPC6 would also cause kidney dysfunction. A single-point mutation in TRPC6, P112Q, is sufficient to lead to focal segmental glomerular sclerosis FSGS (Winn et al., 2005). Other mutations in TRPC6 that lead to nephrosis have been identified (Moller et al., 2007). In addition, it has been found that TRPC6 inactivation or inhibition is involved in the protective role of severe nephrosis (Kim et al., 2018; Lin B. L. et al., 2019). Therefore, increasing interest has been focused on TPRC6 as a potential therapeutic target of acquired kidney diseases.

EMT can be elicited in fibrotic kidney after unilateral ureteral obstruction (Iwano et al., 2002; Klahr and Morrissey, 2003). Inhibition of TRPC6 ameliorates fibrotic damage and contributes to kidney protection (Wu et al., 2017). Yet, the relationship between TRPC6 and EMT in kidney fibrosis is poorly understood. Moreover, the vast majority of studies on fibrosis of EMT were conducted on the animal models and cell lines, while few studies have been performed on the primary culture cells. Therefore, we aimed to study the important role of TRPC6 in the process of EMT after fibrotic injury in animal models and primary TEC.

In the current study, we found that TRPC6 knockout could alleviate kidney tubulointerstitial fibrosis mediated by EMT after unilateral ureteric obstruction (UUO) *in vivo*. We also found that EMT is more down-regulated in primary TEC induced by TGF-β1 from TRPC6^−/−^ mice than from the wild-type (WT) mice *in vitro*. The pathways of the AKT-mTOR and ERK1/2 were both activated in the obstructed kidney and the primary TEC treated with TGF-β1, and down-regulation of AKT-mTOR and ERK1/2 signaling pathways were found in TRPC6^−/−^ mice. We thus hypothesized that TRPC6 knockout may inhibit EMT to ameliorate the kidney injury by down-regulating the AKT-mTOR and ERK1/2 signaling pathways.

## Materials and Methods

### Chemicals and Reagents

Sources of antibodies and reagents were as follows:

Anti-AKT (Cell Signaling Technology, Danvers, MA, USA), anti-p-AKT (Ser473) (Cell Signaling Technology, Danvers, MA, USA), anti-p-ERK1/2 (Cell Signaling Technology, Danvers, MA, USA), anti-ERK1/2 (Cell Signaling Technology, Danvers, MA, USA), anti-p-mTOR (Cell Signaling Technology, Danvers, MA, USA), anti-mTOR (Cell Signaling Technology, Danvers, MA, USA), anti-E-cad (Cell Signaling Technology, Danvers, MA, USA), anti-Cadh16 (Proteintech, Chicago, Illinois, USA), anti-snail1(Cell Signaling Technology:3879s), anti-α-SMA (Boster, Wuhan, Hubei, China), anti-AQP1 (Proteintech, Chicago, Illinois, USA), anti-ATP (Proteintech, Chicago, Illinois, USA), anti-TGF-β1 (Proteintech, Chicago, Illinois, USA), anti-GAPDH (Proteintech, Chicago, Illinois, USA), anti-TRPC6 (Alomone, Jerusalem, Israel), anti-TRPC3 (Alomone:ACC-016), anti-mouse IgG (KeRui, Wuhan, Hubei, China), anti-rabbit IgG antibody (KeRui, Wuhan, Hubei, China), recombinant human TGF-β1 (Cell Signaling Technology, Danvers, MA, USA), HYP9 (MedChem, Shanghai, China), and type-2 collagenase (Worthington Biochemical Corporation, Lakewood, Colorado, USA).

DMEM/f12 and FBS were purchased from Invitrogen (Chicago, California, USA). The whole sagittal section of the kidney was scanned by Biossci Biotechnology Company (Wuhan, Hubei, China).

### Mice Models

TRPC6^−/−^ mice on a 129SvEv background were reconstituted at the Comparative Medicine Branch (CMB) of the National Institute of Environmental Health Sciences (NIEHS), North Carolina, USA. WT mice, which served as controls for the knockout mice, were also obtained from NIEHS. Mice were permitted *ad libitum* access to food and water. Mice were kept on a 12-h light/12-h dark cycle in a temperature-controlled room. The protocols were conducted on adult male 8–10-week-old TRPC6^−/−^ mice weighing 20–25 g and their age-matched WT 129SvEv controls. The experiments were carried out in strict accordance with the standard biosecurity and institutional safety procedures of the Laboratory of Huazhong University of Science and Technology. The animal study was reviewed and approved by the Institutional Animal Care and Use Committee at Tongji Medical College, Huazhong University of Science and Technology.

### Experimental Unilateral Ureteric Obstruction

Animals were anesthetized with chloral hydrate before surgery. The surgery of UUO was performed as reported previously (Pang et al., 2009, 2010). Briefly, the left ureter was exposed via a midline abdominal incision under general anesthesia, ligated in two places using 4–0 surgical silk, and cut off between two ligatures. The right ureter was kept intact. In the study, the obstructed left kidney was used for fibrosis analysis, and the contralateral, non-obstructed kidney was used as controls (Masterson et al., 2004). UUO mice were sacrificed on days 3, 7, or 15 after UUO. Kidneys were immediately removed, decapsulated, and cut into small pieces for subsequent protein studies or stored at −80°C. One portion of the kidneys was collected and fixed in 4% neutral-buffered formalin for subsequent histologic and immunohistochemical staining.

### Isolation and Primary Culture of Kidney TEC

Under sterile conditions, primary TEC were extracted and cultured from age-matched male mice on the basis of established culture methods in our lab (Barrallo-Gimeno and Nieto, 2005; Hou et al., 2018). Mice were sacrificed by cervical dislocation, and kidney tissues were collected. After carefully removing the capsule, the cortical tissues were isolated from the kidney, cut into small pieces, and mixed with the type-2 collagenase [DS with 0.1% (wt/vol)] to fully digest in a shaking temperature-controlled water bath kettle for 15 min about three–four times at 37°C. After removal of the supernatant, digestive liquid was added into an equal volume of pre-cold DMEM/F12 supplemented with 10% FBS to terminate digestion and then filtered by two nylon sieves (pore sizes 180 and 75 μm, Bio-Swamp, Wuhan, Hubei, China). The kidney tubular fragments were collected from the 75-μm sieve and resuspended in culture medium after centrifugation for 5 min at 500 g. The culture medium contained DMEM/F12 supplemented with 10% FBS, 1% penicillin/streptomycin, HEPES 15 mM, insulin 10 μg/ml, L-glutamine 2.5 mM, selenium 5 μg/ml (Sigma, St. Louis, Missouri, USA), transferrin 5.5 μg/ml, and sodium pyruvate 0.55 mM (Bio-Swamp, Wuhan, Hubei, China). The fragment tissues were maintained in a standard humidified incubator (Thermo Fisher Scientific, Waltham, MA, USA) at 37°C and in 5% CO_2_-95% air, with culture medium being changed initially at day 3 and subsequently every 2 days. About 5 days later, primary outgrowth cells from tubular fragment were developed as a confluent monolayer and trypsinized for following experiments.

Occasionally, the fragments were seeded onto polylysine-coated glass slides for following cell immunofluorescence analysis.

### Cell Culture and Treatments

Primary TEC were cultured in DMEM/F12 supplemented with 10% FBS and other nutrient factors. After the standard culture of 3 days, the TEC were treated with 5 ng/ml TGF-β1 for 72 h to induce EMT i*n vitro* (Zhou T. et al., 2018). Culture medium with TGF-β1 was replaced every 2 days, and 10 Mm of HYP9 was added into the culture medium for 1.5 h prior to the treatment of TGF-β1 in experiments (Zhou L. F. et al., 2018).

### Histology and Histopathology

Morphology changes of the kidney were examined by hematoxylin and eosin (HE), Masson, and Sirius Red staining. Kidneys were collected and fixed with 4% paraformaldehyde, dehydrated by sucrose, and embedded in OCT compound, then sectioned in 6-μm thickness. The kidney cryosections were washed three times with PBS at room temperature and stained, respectively, with relevant reagents of HE, Masson, and Sirius red by standard protocol. The staining sections were detected by an optical microscope (Olympus, Tokyo, Japan) at ×200 magnification to analyze the interstitial fibrosis of UUO. Kidney injury was confirmed by interstitial fibrosis and tubular damage, which were defined by the structure, integrity of the basal membrane, and of the brush border and the dimension, compared to the healthy kidney tubule.

### Immunofluorescence Staining

Immunofluorescence analysis was conducted according to the standard protocol. For immunofluorescence staining, 4% paraformaldehyde-fixed, sucrose-dehydrated, and OCT-embedded kidney tissues were sectioned to 6-μm thick. Primary TEC adhered to coverslips were fixed with 4% paraformaldehyde for 15 min at room temperature and immersed in 0.5% Triton X-100 for 15–30 min with the same operation of kidney cryosections. To detect the expression of EMT markers and the aquaporins of kidney tubule epithelial cell, kidney cryosections and pre-treated primary TEC adhered to coverslips were incubated at 37°C for 1 h in a humidified chamber with relevant primary antibody (dilution 1:50) of E-cad, Cadh16, α-SMA, snail1, and so on. Samples were then incubated with FITC-conjugated anti-mouse or rabbit secondary antibody (1:200) for 2 h at room temperature. Immunolabelled kidney cells were counterstained with DAPI (Sigma) for 10 min to show nuclear morphology. Stained slides were viewed and scanned at ×200 magnification by a confocal microscope (Olympus, Tokyo, Japan).

### Western Blot Analysis

Fresh kidney samples were solubilized with the lysis buffer of RIPA containing 100 mg/ml PMSF and 1% protease inhibitor cocktail on ice for 30 min, while TEC were solubilized in a lysis mixture containing 50 mM Tris-HCl (pH 6.8), 1 mM PMSF, 1 mM EDTA, 150 mM NaCl, and 1% NP-40 and incubated on ice for 30 min. The tissue and cell lysates were centrifuged at 12,000 g for 15 min at 4°C. After the protein concentration was assessed by BCA protein assay kit, the supernatants were mixed with 4 × SDS sample buffer, boiled for 10 min at 95°C, separated through 8–12% SDS–PAGE gels, and then transferred to a PVDF membrane (Roche, USA) by electroblotting in mini trans-blot cell (Bio-Rad). After being blocked in TBS-Tween buffer consisting of 5% (*w*/*v*) fat-free, dried milk for 30 min, the membrane was incubated with the appropriate primary antibodies (diluted 1:1,000) in skim milk at 4°C overnight and then incubation with the relevant HRP-conjugated secondary antibodies (diluted 1:3,000) in TBST followed. Finally, each protein band was visualized with Pierce ECL (Thermo Fisher Scientific, USA) reagents. Molecular weights were confirmed by comparison with pre-stained SDS–PAGE molecular weight standards (Thermo Fisher Scientific, USA). Densitometry of the western blots was analyzed using the Quantity One software (Bio-Rad, Hercules, CA, USA). Expression of EMT markers was normalized to GAPDH, and phospho-ERK1/2, p-AKT, and p-mTOR were normalized to total ERK1/2, AKT, and mTOR.

### Statistical Analyses

All experiments were performed in triplicate at least three times, and the data results were expressed as mean ± sem (standard error of the mean). The statistical differences between groups were assessed by using one-way analysis of variance (ANOVA) depending on the number of comparisons being made and the data distribution. When *p*-values were <0.05, the differences were considered significant.

## Results

### Kidney Fibrosis Increases TRPC6 Expression and Activates EMT Program

**Figure 1:**
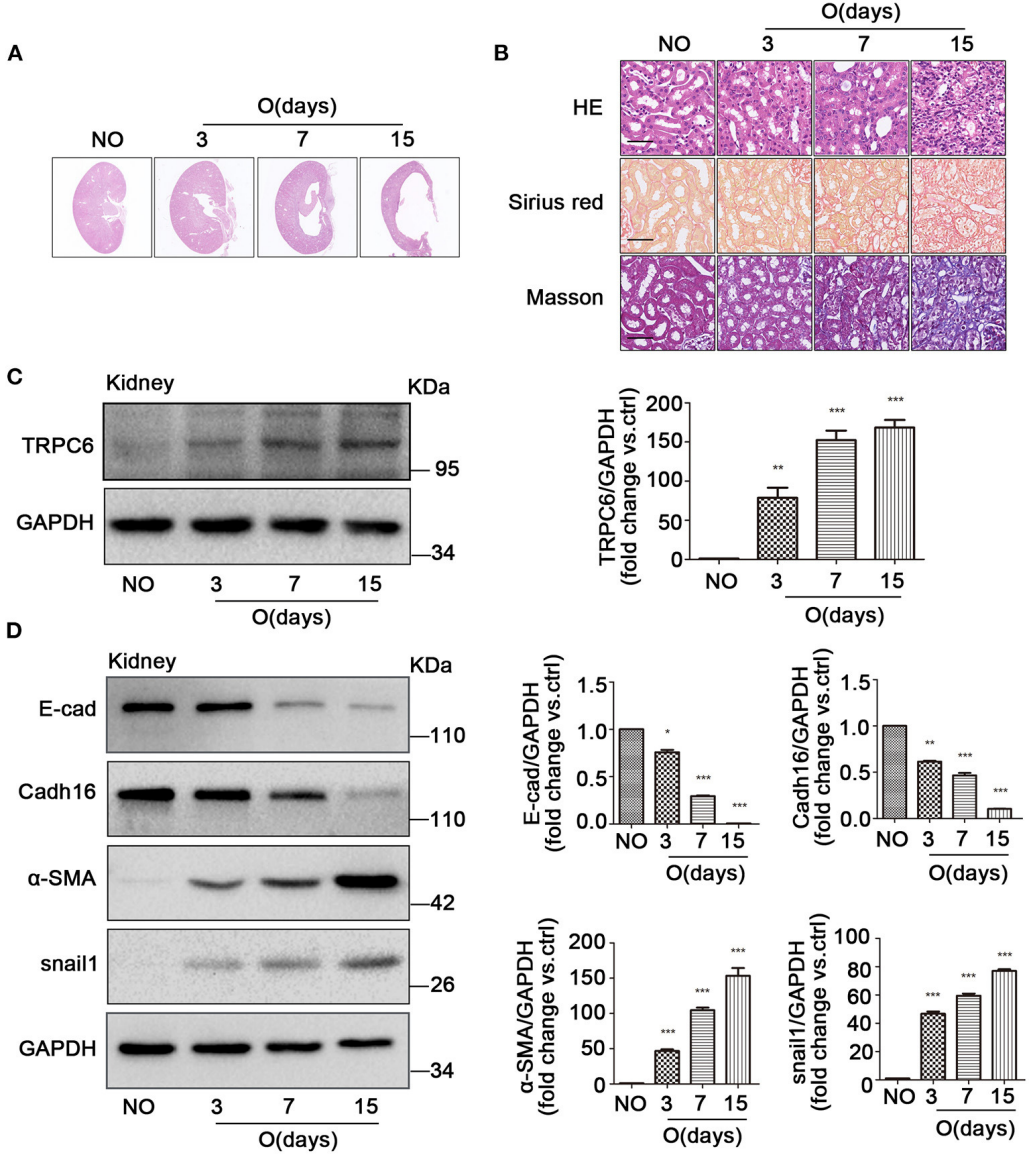
The increase of TRPC6 and activation of EMT in fibrotic kidney tissues. **(A)** Representative scanning images of kidney coronal plane showing the cavity caused by fibrosis injury in the contralateral non-obstructed (NO) and obstructed (O) kidneys from WT mice on days 3, 7, and 15 after UUO. **(B)** Representative images of similar kidney sections stained by HE, Masson, and Sirius red showing the interstitial fibrosis change of kidney tissues from the above groups. Scale bars, 20 μm. **(C)** Expression level of TRPC6 from the above groups detected by western blot. Data are presented as mean ± sem, *n* = 3; an unpaired two-tailed *t*-test was used. **P < 0.01, ***P < 0.001. **(D)** Expression level of EMT markers E-cad, Cadh16, α-SMA, and snail1 from the above groups detected by western blot. Data are presented as mean ± sem, *n* = 3; an unpaired two-tailed *t*-test was used. *P < 0.05, **P < 0.01, ***P < 0.001.

After UUO, fibrotic damages occurred in the obstructed kidney (Martinez-Klimova et al., 2019). Compared to the non-obstructed (NO) contralateral kidneys in UUO mice, which has a normal kidney structure, an obvious cavity can be observed in the coronal plane of the obstructed (O) kidney, while the cavity size markedly increased with the obstructed time extending after UUO (Figure 1A). A mass of extracellular matrix is produced in the process of kidney fibrosis, which is characteristic of fibrosis (Duffield, 2014). Therefore, HE, Masson, and Sirius red staining were conducted to explore histopathologic changes and accumulation of matrix and fibers related to fibrosis. The images revealed that a significant tubular impairment became worse by HE staining, and a higher degree of tubulointerstitial fibrosis was increased in the obstructed kidneys of WT mice, on days 3, 7, and 15 after UUO, relative to the non-obstructed kidney by Masson staining (collagen fibers, blue) and by Sirius red staining (collagen fibers, red) (Figure 1B). In order to better understand whether TRPC6 plays a key role in kidney fibrosis, we inspected the expression level of TRPC6 by western blot. Results showed that TRPC6 had a time-dependent increase in obstructed kidneys (Figure 1C), while the expression of TGF-β1, an important inducer to fibrosis, was also increased in the fibrotic kidney after UUO (Supplementary Figure 1A). So as to determine the participation of EMT in kidney fibrosis after UUO, EMT markers, such as E-cad, Cadh16, α-SMA, and snail1, were examined at the protein level by western blot. The expression levels of E-cad and Cadh16 were down-regulated, and α-SMA and snail1 were markedly upregulated in obstructed kidneys induced by UUO compared with the non-obstructed group (Figure 1D). These phenomena suggest that there may exist some connections between TRPC6 and EMT activation in kidney fibrosis.

### TRPC6 Knockout Partially Inhibits EMT to Alleviate Kidney Injury

**Figure 2:**
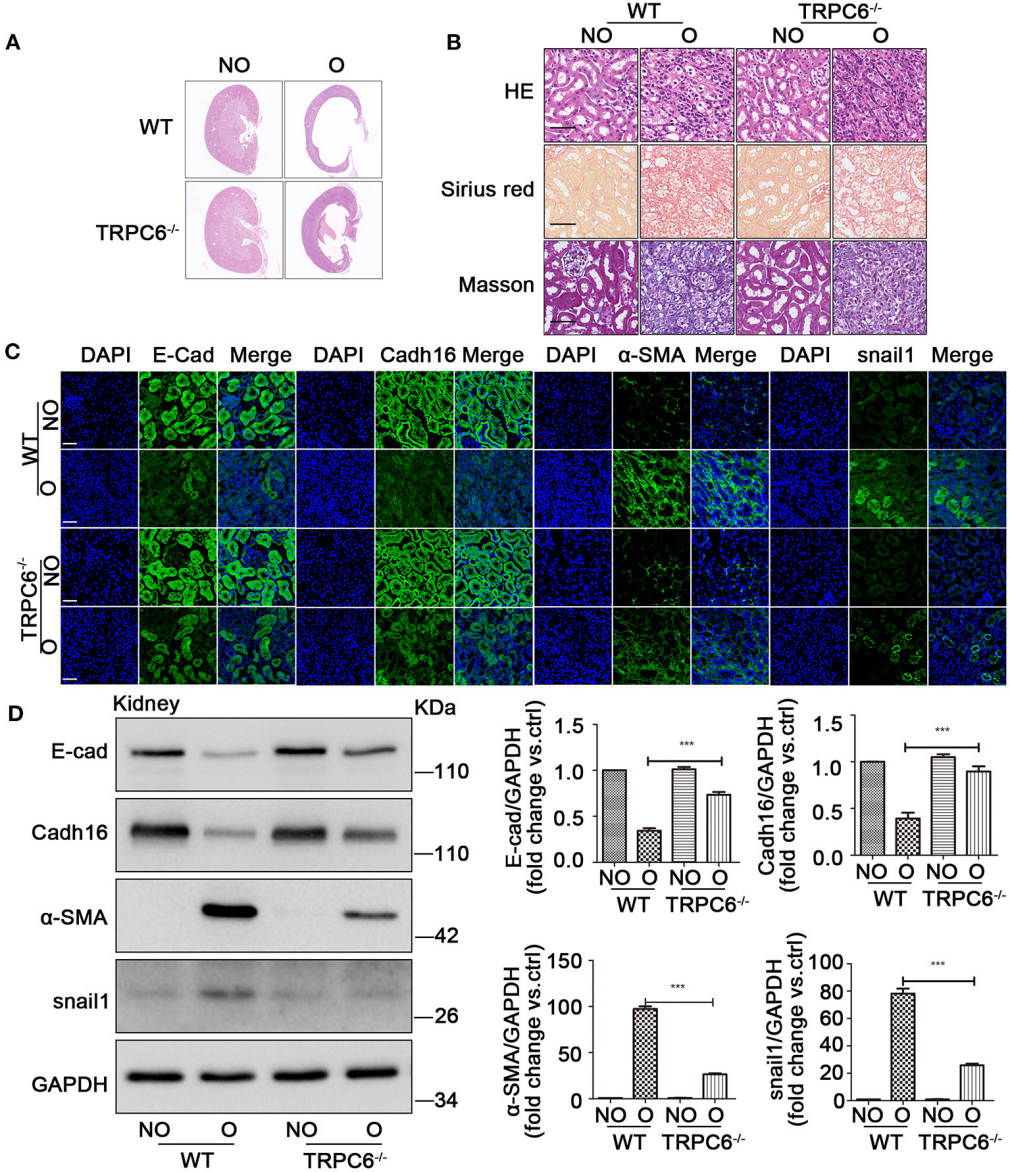
TRPC6 knockout partially inhibits EMT to alleviate the kidney injury. **(A)** Representative scanning images of kidney coronal plane showing the cavity caused by fibrotic injury in NO and O kidneys from TRPC6 knockout and WT mice on day 15 after UUO. **(B)** Representative images of kidney sections stained by HE, Masson, and Sirius red showing interstitial fibrosis change of kidney tissues from the above groups. Scale bars, 20 μm. **(C)** Expression level of EMT markers (green) E-cad, Cadh16, α-SMA, and snail1 as well as nuclear stain DAPI (blue) from the above groups detected by immunofluorescence analysis. Scale bars, 20 μm. **(D)** Expression level of EMT markers E-cad, Cadh16, α-SMA, and snail1 in NO and O kidneys from the above groups detected by western blot. Data are presented as mean ± sem, *n* = 3; an unpaired two-tailed *t*-test was used. ***P < 0.001.

To investigate the relationship between TRPC6 and EMT in kidney fibrosis, the TRPC6^−/−^ mice were used in the study. On day 15 after UUO, the size of the cavity in obstructed kidneys became overtly smaller in TRPC6^−/−^ mice compared to WT mice (Figure 2A), and the swelling of the obstructed kidney was obviously alleviated (Supplementary Figure 1B). The result showed that TRPC6 knockout could improve kidney health obviously in obstructed kidney. Then, the data of HE, Masson, and Sirius red staining also revealed a lower degree of injury and tubulointerstitial fibrosis in TRPC6^−/−^ mice after UUO (Figure 2B). The tubular impairment was obviously alleviated and the accumulation of collagen fibers decreased apparently. In order to explore the EMT activation in kidney fibrosis, we examined the expression of EMT markers through immunofluorescent staining and western blot. The staining results showed that TRPC6 knockout could partially inhibit EMT and have an anti-fibrosis effect in obstructed kidney induced by UUO (Figure 2C). The decrease of E-cad and Cadh16 expression was prevented in obstructed kidneys of TRPC6^−/−^ mice compared to that of WT mice, while the increase of α-SMA and snail1 expression was also inhibited in TRPC6^−/−^ mice. To further confirm the result, the levels of EMT markers were detected by western blot (Figure 2D). All of the results showed that TRPC6 knockout plays a protective role along with the inhibition of EMT in kidney fibrosis.

### TRPC6 Increases in TEC After TGF-β1 Treatment Along With the Activation of EMT

**Figure 3:**
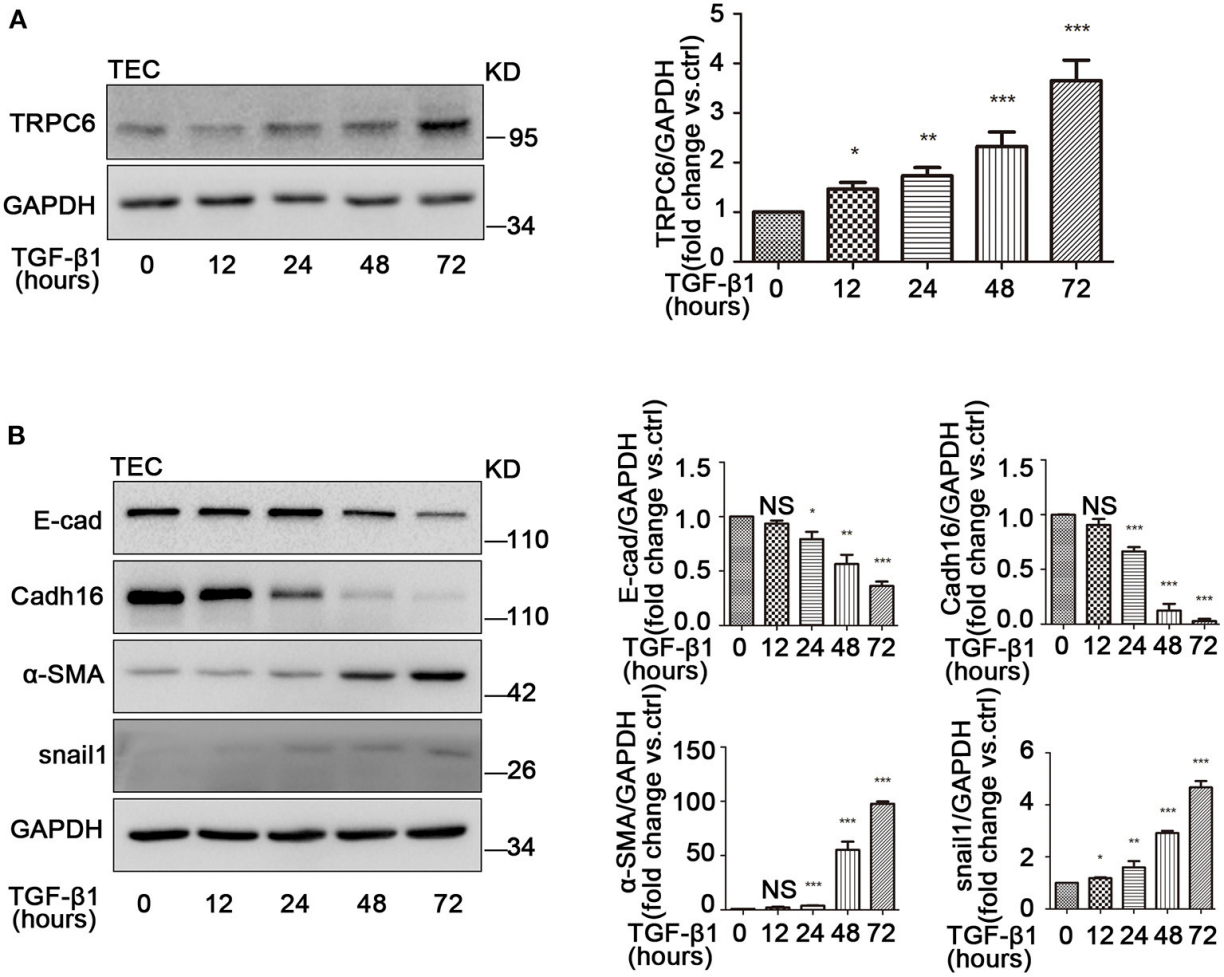
The increase of TRPC6 and activation of EMT in primary TEC stimulated with TGF-β1. **(A)** Expression of TRPC6 in primary TEC without and with TGF-β1 (5 ng/ml in 20 mM citric acid, pH 3.0) stimulation at 12, 24, 48, and 72 h detected by western blot. Data are presented as mean ± sem, *n* = 3; an unpaired two-tailed *t*-test was used. *P < 0.05, **P < 0.01, ***P < 0.001. **(B)** Relative expression of the EMT markers including E-cad, Cadh16, α-SMA, and snail1 in primary TEC with and without TGF-β1 stimulation for 72 h detected by western blot. Data are presented as mean ± sem, *n* = 3; an unpaired two-tailed *t*-test was used. NS, not significant; *P < 0.05, **P < 0.01, ***P < 0.001.

Several studies have demonstrated that TGF-β1 is the master inducer of EMT in kidney fibrosis (Meng et al., 2016); therefore, we next examined the change of EMT in primary TEC after stimulation with TGF-β1. Primary TEC were treated with TGF-β1 for different times (12, 24, 48, and 72 h) at a concentration of 5 ng/ml and had an obvious phenotype change with the extended stimulation time. Under normal culture conditions, the primary TEC grew from the kidney tissue and fused to monolayer on day 5 (Supplementary Figure 1C). After treatment with TGF-β1 for 72 h, the cells exhibited morphology changes (Supplementary Figure 1D). It is known that TRPC3, TRPC6, and TRPC7 are in a homologous group and always work together in various pathological courses (Chen et al., 2017; He et al., 2017). Due to the lack of TRPC7 expression in the kidney (Liu et al., 2017), we examined the expression levels of TRPC3 and TRPC6 in primary TEC induced by TGF-β1. We discovered that the level of TRPC6 expression was enhanced, but the level of TRPC3 expression had no obvious change in primary TEC stimulated by TGF-β1 (Supplementary Figure 2A). In our lab, we had previously confirmed that TEC from TRPC6^−/−^ mice lack TRPC6 expression but had normal TRPC3 isoforms compared with TEC from the control group, and TRPC6 has a functional significance in TEC as a store-operated Ca^2+^ channel in primary TEC from WT mice (Hou et al., 2018). Western blot showed that TRPC6 expression increased gradually in primary TEC induced by TGF-β1 (Figure 3A). This highlighted that TRPC6 played a key role in primary TEC from WT mice treated with TGF-β1. It is also well-known that TGF-β1 is a common stimulator of EMT induction in cell lines. Next, we investigated whether the EMT was activated in primary TEC stimulated by TGF-β1. The markers of EMT, including E-cad, Cadh16, α-SMA and snail1, were examined by western blot. The data showed that the time-course of E-cad and Cadh16 expression decreased distinctly, while that of α-SMA and snail1 increased markedly (Figure 3B). This demonstrated that EMT was also activated in the primary TEC stimulated with TGF-β1 in line with the result of animal model. These results suggested that TRPC6 has a close link with EMT activation in primary TEC after treatment.

### TRPC6 Knockout Partially Inhibits EMT Induced by TGF-β1 in Primary TEC

**Figure 4:**
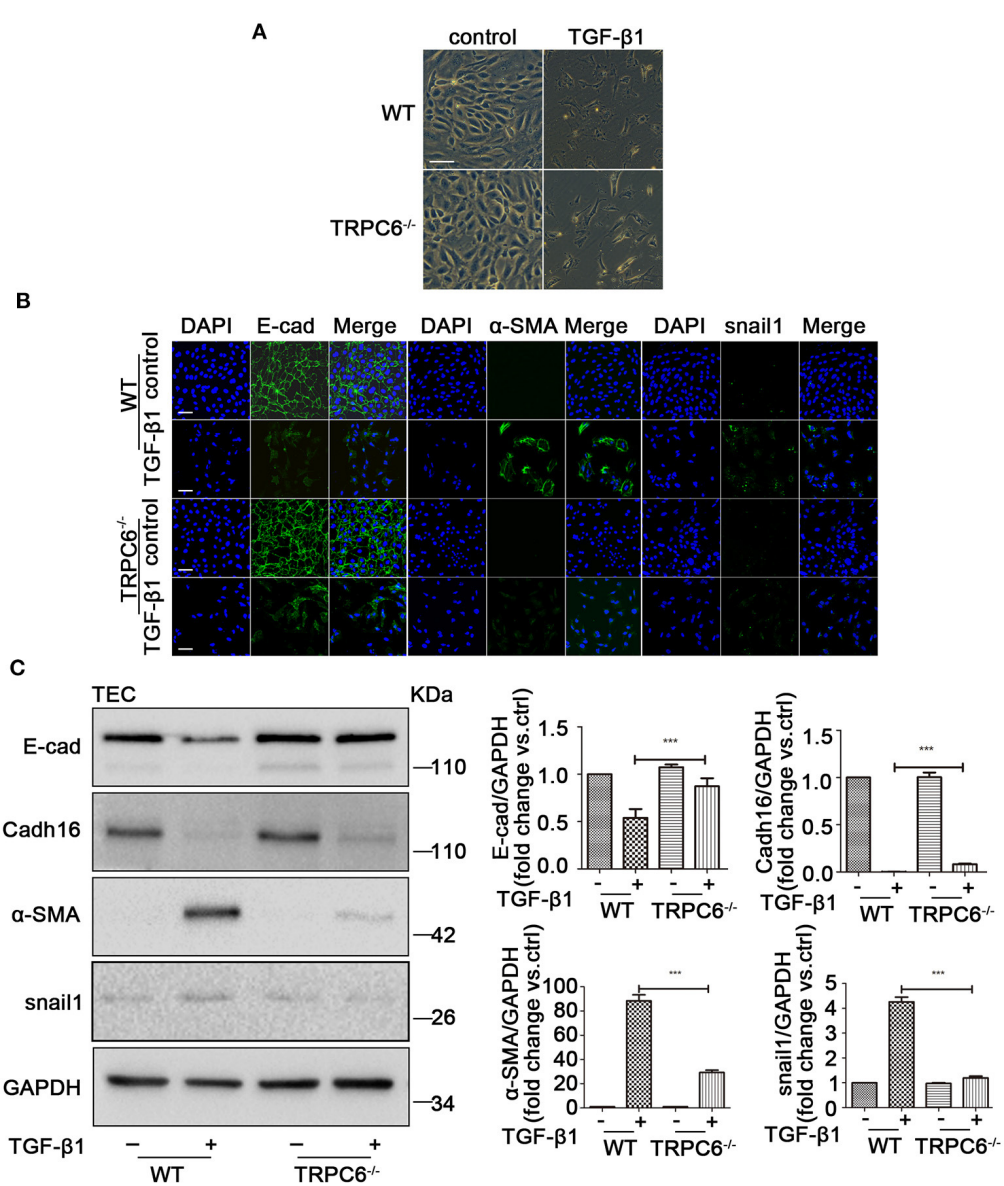
The process of EMT was inhibited in primary TEC stimulated with TGF-β1 inTRPC6^−/−^ mice. **(A)** Morphological changes of primary TEC from WT and TRPC6^−/−^ mice with and without TGF-β1 stimulation for 72 h. **(B)** Expression level of EMT markers (green) E-cad, α-SMA, and snail1 as well as nuclear stain DAPI (blue) from the above groups detected by immunofluorescence analysis. Scale bars, 20 μm. **(C)** Expression level of EMT markers E-cad, Cadh16, α-SMA, and snail1 from the above groups detected by western blot. Data are presented as mean ± sem, *n* = 3; an unpaired two-tailed *t*-test was used. ***P < 0.001.

To explore the anti-fibrosis effect of TRPC6 in EMT-mediated kidney fibrosis, primary TEC of WT and TRPC6^−/−^ mice were stimulated with 5 ng/ml TGF-β1 for 72 h to mimic obstructed injury *in vitro*. The TEC from the WT mice displayed typical cobblestone-like morphology without stimulation of TGF-β1 under the inverted microscope, exhibited polygon or fusiform morphology, and even stretched out some long pseudopods after treatment with TGF-β1 for 72 h (Figure 4A). The TEC from the TRPC6^−/−^ mice, however, showed a lesser degree of change in the same treatment condition than TEC from the WT mice, and it was rather difficult to see the pseudopods around the cells in the TRPC6 knockout group. To explore the phenomena further, immunofluorescent staining was used to test the expression levels of EMT markers. As expected, it showed that the activation of EMT in TEC from TRPC6^−/−^ mice was markedly prevented. The decrease of E-cad expression and increase of α-SMA and snail1 in treatment groups of TEC from TRPC6^−/−^ mice were reversed compared to that of WT mice (Figure 4B). Cadh16 was lost in the original microenvironment of the kidney in cultured primary TEC, so it cannot be inspected by immunofluorescent staining. As further evidence, the expression levels of EMT markers were detected by western blot, and a consistent result was seen (Figure 4C). The results further confirmed that TRPC6 knockout has a protective effect with the inhibition of EMT in TEC treated with TGF-β1.

### TRPC6 Knockout Negatively Regulates the AKT-mTOR and ERK1/2 Signaling Pathways

**Figure 5:**
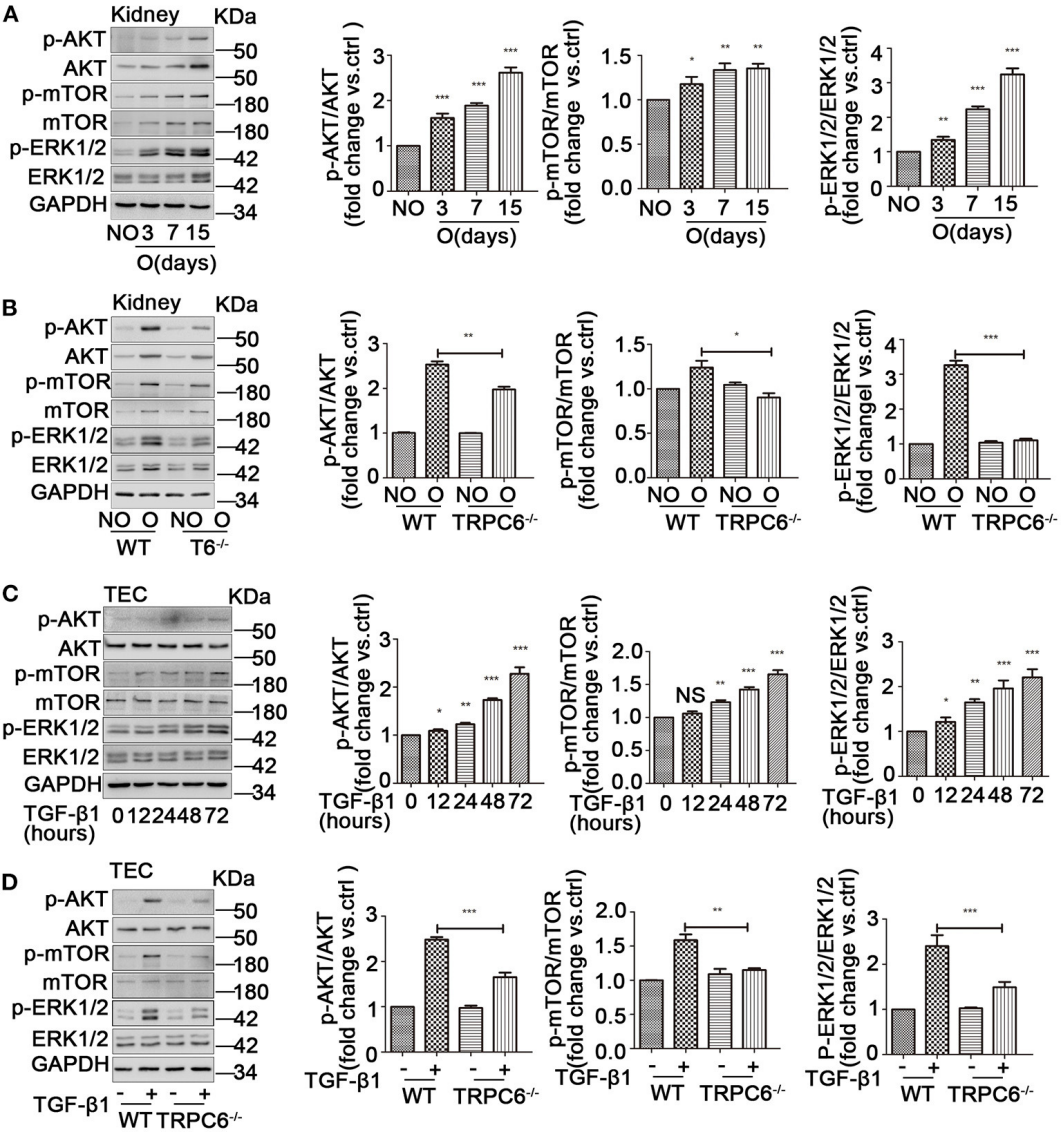
The activation of AKT-mTOR and ERK1/2 signaling pathways was down-regulated in TRPC6^−/−^ mice. **(A)** Expression level of the phosphorylated and total protein of AKT, mTOR, and ERK1/2 in NO and O kidneys from WT mice on days 3, 7, and 15 after UUO detected by western blot. Data are presented as mean ± sem, *n* = 3; an unpaired two-tailed *t*-test was used. *P < 0.05, **P < 0.01, ***P < 0.001. **(B)** Expression level of the phosphorylated and total protein of AKT, mTOR, and ERK1/2 in NO and O kidneys from TRPC6^−/−^ and WT mice on day 15 after UUO detected by western blot. Data are presented as mean ± sem, *n* = 3; an unpaired two-tailed *t*-test was used. *P < 0.05, **P < 0.01, ***P < 0.001. **(C)** Expression level of the phosphorylated and total protein of AKT, mTOR, and ERK1/2 in primary TEC without and with TGF-β1 stimulation at 12, 24, 48, and 72 h detected by western blot. Data are presented as mean ± sem, *n* = 3; an unpaired two-tailed *t*-test was used. NS, not significant; *P < 0.05, **P < 0.01, ***P < 0.001. **(D)** Expression level of the phosphorylated and total protein of AKT, mTOR, and ERK1/2 in primary TEC from WT and TRPC6^−/−^ mice with and without TGF-β1 stimulation for 72 h detected by western blot. Data are presented as mean ± sem, *n* = 3; an unpaired two-tailed *t*-test was used. **P < 0.01, ***P < 0.001.

AKT kinase is likely a critical regulator in cellular activity including cell proliferation, differentiation, and apoptosis. Moreover, AKT/mTOR is a vital signaling pathway mediated many physiological and pathological processes of the kidney (Yoo et al., 2011; Yang et al., 2015). In addition, it was previously reported that ERK1/2 could be activated and have an important role in the program of fibrosis. We presumed that an AKT/mTOR-related or/and ERK1/2-related response could be activated in UUO-induced kidney fibrosis and cultured primary TEC treated with TGF-β1. As expected, the phosphorylation ratio of AKT (Ser473), mTOR (Ser2448), and ERK1/2 was increased during the process of EMT activation in the obstructed kidneys (Figure 5A) and the primary TEC stimulated by TGF-β1 (Figure 5C). Additionally, the obstructed kidney of TRPC6^−/−^ mice showed lower ratio of p-AKT, p-mTOR, and p-ERK1/2 than that of WT mice (Figure 5B). This result suggested the AKT-mTOR or/and ERK1/2 signaling pathways were activated in kidney fibrosis and were down-regulated in TRPC6^−/−^ mice. TRPC6 knockout may alleviate kidney fibrosis through negative regulation of AKT-mTOR or/and ERK1/2 signaling pathways. In order to ascertain whether the hypothesis is the same with the EMT activation of primary TEC treated by TGF-β1, we examined the protein expression of related signaling molecules by western blot. We found that the ratio of p-AKT, p-mTOR, and p-ERK1/2 in treated TEC from TRPC6^−/−^ mice was markedly lower than their WT counterparts (Figure 5D). This observation is in line with the consequence of TRPC6^−/−^ mice models by UUO.

### TRPC6 Knockout Improves the Loss of Na^+^/K^+^-ATPase and AQP1 in the Kidney

**Figure 6:**
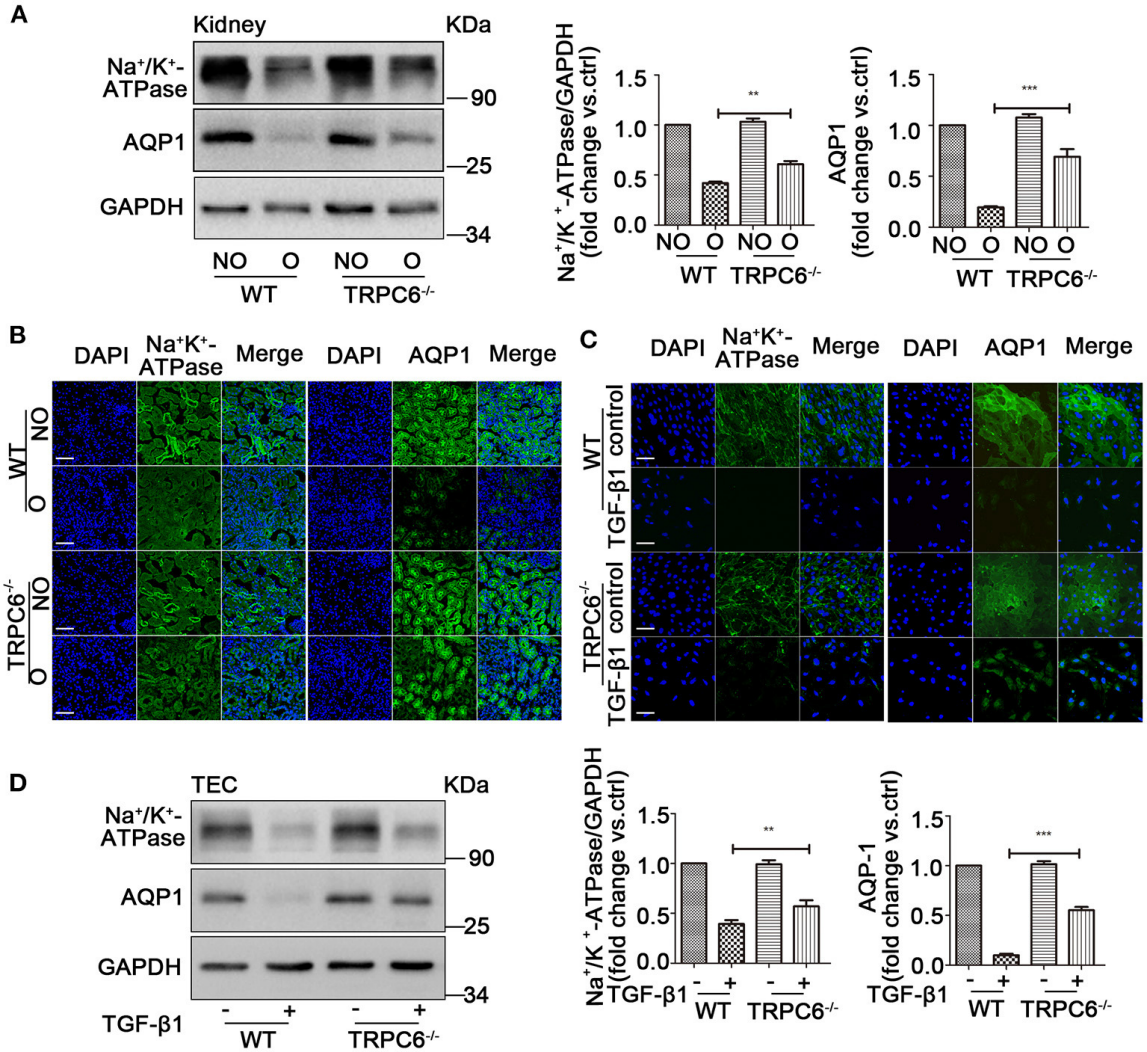
TRPC6 knockout improves the loss of channel proteins on kidney tubulars and TEC. **(A)** Expression level of Na^+^/K^+^-ATPase and AQP1 in NO and O kidneys from TRPC6 knockout and WT mice on day 15 after UUO detected by western blot. Data are presented as mean ± sem, *n* = 3; an unpaired two-tailed *t*-test was used. **P < 0.01, ***P < 0.001. **(B)** Expression level of Na^+^/K^+^-ATPase and AQP1 (green) as well as nuclear stain DAPI (blue) in NO and O kidneys from TRPC6^−/−^ and WT mice on day 15 after UUO detected by immunofluorescent staining. Scale bars, 20 μm. **(C)** Expression level of Na^+^/K^+^-ATPase and AQP1 (green) as well as nuclear stain DAPI (blue) in primary TEC with and without TGF-β1 stimulation for 72 h detected by immunofluorescent staining. Scale bars, 20 μm. **(D)** Expression level of Na^+^/K^+^-ATPase solute transporter and AQP1 in primary TEC with and without TGF-β1 stimulation for 72 h detected by western blot. Data are presented as mean ± sem, *n* = 3; an unpaired two-tailed *t*-test was used. **P < 0.01, ***P < 0.001.

The realization of kidney function relies on the channel protein, and the loss of channel protein would significantly affect normal filtration function and lead to kidney dysfunction, which is harmful to our engine body. A specific reduction in the expression of Na^+^/K^+^-ATPase (ion channel protein) and aquaporin1 (AQP1) is shown in obstructed kidney tissues after UUO compared to contralateral kidneys, as well as an obvious improvement in TRPC6^−/−^ mice compared to WT mice, which is evident by western blotting and immunofluorescent staining (Figures 6A,B). In order to verify the assumption that the effect is no different in culture TEC, the same tests were done by western blot and immunofluorescent staining. A consistent result was detected in TEC treated with TGF-β1 compared to controls from WT mice, and TRPC6^−/−^ mice showed a reversal of the decrease compared to WT mice (Figures 6C,D). These results, therefore, show that TRPC6 knockout would ameliorate kidney dysfunction caused by kidney fibrosis.

## Discussion

Fibrosis is normally beneficial as a tissue repair process; however, if excessive, it leads to relentless tissue damage and failure of organ function in severe cases. In the process of kidney fibrosis, the myofibroblast plays a central role and its aberrant growth contributes to the over-production of extracellular matrix (Liu, 2004; Neilson, 2006; Wynn, 2008; Zubair and Ahmad, 2019). Generally, the myofibroblast is derived from the proliferation and differentiation of the fibroblast. The TEC, another important origin of myofibroblast, has been of interest to researchers recently. That epithelial cell could translate to the myofibroblast by EMT. Studies have found that EMT is of profound significance in fibrosis and pervasive in different organs, such as the lungs, lens, liver, and kidney (Liang et al., 2014; Kong et al., 2015; Yang et al., 2019; Yao et al., 2019). Furthermore, EMT has been observed to improve the onset of kidney fibrosis, and fibrotic damages could be alleviated by repressing the process of EMT (Du et al., 2012; Geng et al., 2019). Clinical studies have suggested that EMT, as the early onset element of interstitial fibrosis in the kidney, is confirmed in diabetic patients (Hills and Squires, 2011; Carew et al., 2012). The direct role of EMT of TEC in kidney fibrosis remains controversial. In 2015, Grande and Lovisa et al. independently pinpointed a partial epithelial-to-mesenchymal transition as a mechanism driving the development of kidney fibrosis. They observed that TEC undergo an incomplete EMT; TEC with partial EMT remain attached in the basement of membrane and promote kidney fibrosis via following three mechanisms. In cell-autonomous manner, a partial EMT causes cell-cycle arrest, halting further proliferation and repair; the partial EMT secretes growth factors such as TGF-β and drives myofibroblast proliferation; pathological secretome promotes kidney chronic inflammation(Grande et al., 2015; Lovisa et al., 2015).

TRPCs have a close correlation with kidney disease and are located in the glomeruli and tubules of the kidney (Woudenberg-Vrenken et al., 2009; Liu et al., 2010, 2011). More specifically, TRPC3 and TRPC6 are homologous in structure, but compose heteromultimers in different cell types. TRPC6 is widely present in podocytes, vascular smooth muscle and endothelial cells, mesangial cells, interstitial fibroblasts, as well as TEC. Recent studies have reported that the expression of TRPC3 and TRPC6 was both increased in UUO kidneys, and inhibition of TRPC6 ameliorates kidney fibrosis and contributes to kidney protection (Wu et al., 2017). Another study showed that TRPC3 was increased in the fibroblasts of obstructed kidney, and TRPC3 knockout could inhibit fibroblast proliferation by regulating the ERK1/2 signaling pathway (Saliba et al., 2015). Of note, we found only TRPC6 was obviously increased in primary TEC treated with TGF-β1 and did not find the increase of TRPC3 (Supplementary Figure 2A). Therefore, we focused on TRPC6 in the study of kidney fibrosis on TEC. Furthermore, a study of transdifferentiation of fibroblast to myofibroblast has shown that TRPC6 promoted the transdifferentiation and loss of TRPC6 impeded the process (Davis et al., 2012). However, whether TRPC6 contributes to the transition of TEC to myofibroblast is not clear. To date, no study uncovered the role of TRPC6 on the EMT in renal fibrosis. In our study, coincident results from previous studies were found. TRPC6 expression increased in the injured kidney after UUO in WT mice compared to the contralateral control, and EMT was activated along with the increase of TRPC6. The results revealed TRPC6 expression was strongly related to the activation of EMT in the obstructed kidney. In order to further determine the role of TRPC6 in the process of EMT, TRPC6^−/−^ mice were introduced in the following study. We found the marker change for EMT was partially reversed in obstructed kidney of TRPC6^−/−^ mice compared to that of WT mice. This result suggested EMT was inhibited by the deletion of TRPC6 in fibrotic kidney. With regard to further investigation of EMT and TRPC6, we then aimed to study the primary TEC from kidney tissue.

UUO, a well-established model, enables the study of kidney fibrosis in different pathologic aspects, especially in EMT (Chevalier et al., 2009). When various types of damages from UUO are experienced, TEC become vulnerable, lose some epithelial features, and acquire mesenchymal characteristics leading to the happening of EMT. In the obstructed kidney, an obvious decrease of epithelial markers E-cad and Cadh16 was observed in the TEC as well as the increase of mesenchymal features such as α-SMA and snail1. We also found that an injury cavity appeared that was caused by rampant kidney fibrosis. Importantly, the size of the cavity became larger along with the days of UUO and became smaller following the decrease of EMT. Furthermore, the promotion of EMT augments the damage of TEC, resulting in the decrease of functional capabilities. The kidney function was valued by examination of Na^+^/K^+^-ATPase and APQ1, which are expressed at the plasma membrane of tubule in the kidney to transport the ion and water (Verkman, 2002). Reduction of the Na^+^/K^+^-ATPase and APQ1 is closely connected with kidney function and has been proposed to promote fibrosis and increase metabolites and uremic toxin (Rajasekaran et al., 2010; Liu et al., 2012; Ito et al., 2013). As Na^+^/K^+^-ATPase and APQ1 are necessary to maintain the morphological structure of TEC and filtration function of kidney, so a sharp decrease of them would cause a more serious effect for our engine body. In our study, the low expression of Na^+^/K^+^-ATPase and APQ1 was found in fibrotic kidney and TGF-β1-induced TEC, which were obviously reversed in TRPC6^−/−^ mice. The results suggested that kidney function was recovered to a certain extent with the inhibition of EMT. Therefore, these results highlight the significance of EMT in the development of kidney fibrosis and that kidney damage was alleviated by the inhibition of EMT.

Recently, a majority of studies about EMT in kidney fibrosis focus on TEC lines such as NRK-52E, HEK293T, and HK-2 (Hu et al., 2018; Zhou T. et al., 2018). Few studies have focused on primary cultured TEC. To further confirm the hypothesis that TRPC6 knockout could alleviate fibrotic damage by inhibition of EMT, most of our work *in vitro* was performed on the primary cultured TEC in parallel with the *in vivo* mice model study. The obvious changes of EMT markers, including the decrease of E-cadherin and Cadh16 along with the increase of α-SMA and snail1, occurred when the primary TEC were stimulated with TGF-β1 for 72 h. For the first time, we have found that TRPC6, not TRPC3, was upregulated in primary TEC treated with TGF-β1 from the WT mice, and the phenotypic changes of EMT were also partially reversed in primary TEC from the TRPC6^−/−^ mice. Immunofluorescence assay showed that primary TEC from the TRPC6^−/−^ mice lack TRPC6 isoforms and had normal TRPC3 expression compared to TEC from the WT mice (Supplementary Figure 2B). Additionally, to address the relationship between TRPC6 and EMT, HYP9, the agonist of TRPC6, was used in primary TEC. We found a promotion of EMT in the HYP9-stimulated group compared to the control group (Supplementary Figure 3A). These results provide powerful evidence that the anti-fibrotic effect of TRPC6 knockout is related to the inhibition of EMT.

AKT, a serine/threonine protein kinase, acts as a downstream effector for PI3K (Cantrell, 2001) and plays a significant role in biological functions by activating its downstream effectors (Bertacchini et al., 2015; Jiang et al., 2015). mTOR is a key regulator and can be activated by phosphorylated-AKT. The activation of AKT-mTOR is found to be important in contributing to the fibrosis in different organs, including the lungs, liver, and heart (Wang et al., 2015; Reilly et al., 2017; Cui et al., 2019; Wan et al., 2019), which can regulate cell growth and promote EMT in the kidney (Yoo et al., 2011; Xuan et al., 2014; Carpenter et al., 2015; Yang et al., 2015). Furthermore, the activation of ERK1/2 also significantly contributes to the onset of EMT (Chiu et al., 2017; Sankpal et al., 2017; Jia et al., 2018; Lee et al., 2019). Several studies have shown that the activation of AKT-ERK1/2 plays an important role in the process of EMT in fibrosis and that EMT was initiated through AKT-ERK1/2 activation (Wang et al., 2016; Qu et al., 2019). In order to determine whether the AKT-mTOR and ERK1/2 pathways would be related to the anti-fibrosis effect of TRPC6 knockout in the kidney, the relevant signal molecules were examined in both kidney tissues and primary TEC. In line with previous statements, our data showed that the ratio of phosphorylated-AKT, mTOR, and ERK1/2 was up-regulated strongly in obstructed kidney and TGF-β1-induced TEC. The expression of total AKT, mTOR, and ERK1/2 was also increased in kidney tissues after UUO, and this is consistent with previous studies (Hanatani et al., 2014; Higgins et al., 2017). The results confirmed that the pathways of AKT-mTOR and ERK1/2 were activated in kidney fibrosis. Furthermore, TRPC6^−/−^ mice showed an obvious down-regulation of AKT-mTOR and ERK1/2 after UUO. Beyond that, we found the up-regulation of AKT-mTOR and ERK1/2 pathways in the HYP9-stimulated group compared to the control group (Supplementary Figure 3B). Therefore, TRPC6 knockout protects the fibrotic kidney through down-regulation of AKT-mTOR and ERK1/2 pathways. Otherwise, EMT was partially inhibited in TRPC6^−/−^ mice and up-regulated along with the positive regulation of AKT-mTOR and ERK1/2 in the HYP9-stimulated group. We, therefore, concluded that TRPC6 knockout may protect the fibrotic kidney by inhibition of EMT through down-regulation of AKT-mTOR and ERK1/2 pathways. Whether other pathways beside AKT-mTOR and ERK1/2, such as Wnt/β-catenin, GSK3β, and Ras, are involved in the regulation of the phenotypic change of EMT by TRPC6 in kidney fibrosis requires further investigation.

In conclusion, we found that EMT was activated in kidney fibrosis both *in vitro* and *in vivo*. Fibrosis damage was alleviated in TRPC6^−/−^ mice after UUO and in primary TEC treated with TGF-β1. Moreover, EMT was partially reversed along with the down-regulation of AKT-mTOR and ERK1/2 in TRPC6^−/−^ mice. Therefore, we proposed that TRPC6 knockout may ameliorate kidney damage by the inhibition of EMT through down-regulating the AKT-mTOR and ERK1/2 pathways.

## Data Availability Statement

The original contributions presented in the study are included in the article/Supplementary Materials, further inquiries can be directed to the corresponding author/s.

## Ethics Statement

The animal study was reviewed and approved by the ethics committee of Tongji Medical College of Huazhong University of Science and Technology.

## Author Contributions

YL, MZ, and YZ designed the research. YZ and NY performed experiments, analyzed data, and wrote the manuscript. AS, QW, WH, XH, and XZ contributed to the interpretation of the results. YL and MZ contributed to the analysis of data and preparation of the manuscript. All authors contributed to revise the article and approved the submitted version.

## Conflict of Interest

The authors declare that the research was conducted in the absence of any commercial or financial relationships that could be construed as a potential conflict of interest.

## Abbreviations

EMT: epithelial–mesenchymal transition
TEC: tubular epithelial cells
UUO: unilateral ureteric obstruction
TRPCs: transient receptor potential channels
E-cad: E-cadherin
Cadh16: Cadherin16
NO: non-obstructed
O: obstructed.
