# Supplementary material for: Transient Receptor Potential Channel 6 Knockout Ameliorates Kidney Fibrosis by Inhibition of Epithelial–Mesenchymal Transition
Source: Front Cell Dev Biol. 2021 Jan 15;8:602703. doi: 10.3389/fcell.2020.602703 (PMC7843578; doi:10.3389/fcell.2020.602703)

**Supplementary Figures**

**Supplementary Figure S_1_ The injury in kidney after UUO and primary TEC after TGF-β1 treatment.** **(A)** Expression level of TGF-β1 in NO and O kidneys from WT mice on day3, day7 and day15 after UUO. **(B)** Representative images of post-operation kidney showing the injury in NO and O kidneys from TRPC6 knockout and WT mice on day15 after UUO. **(C)** Representative figures of changes during the culture process of primary TEC. **(D)** Representative images of the phenotype change in culture TEC with or without TGF-β1 stimulation at 12 h, 24 h, 48 h and 72 h.


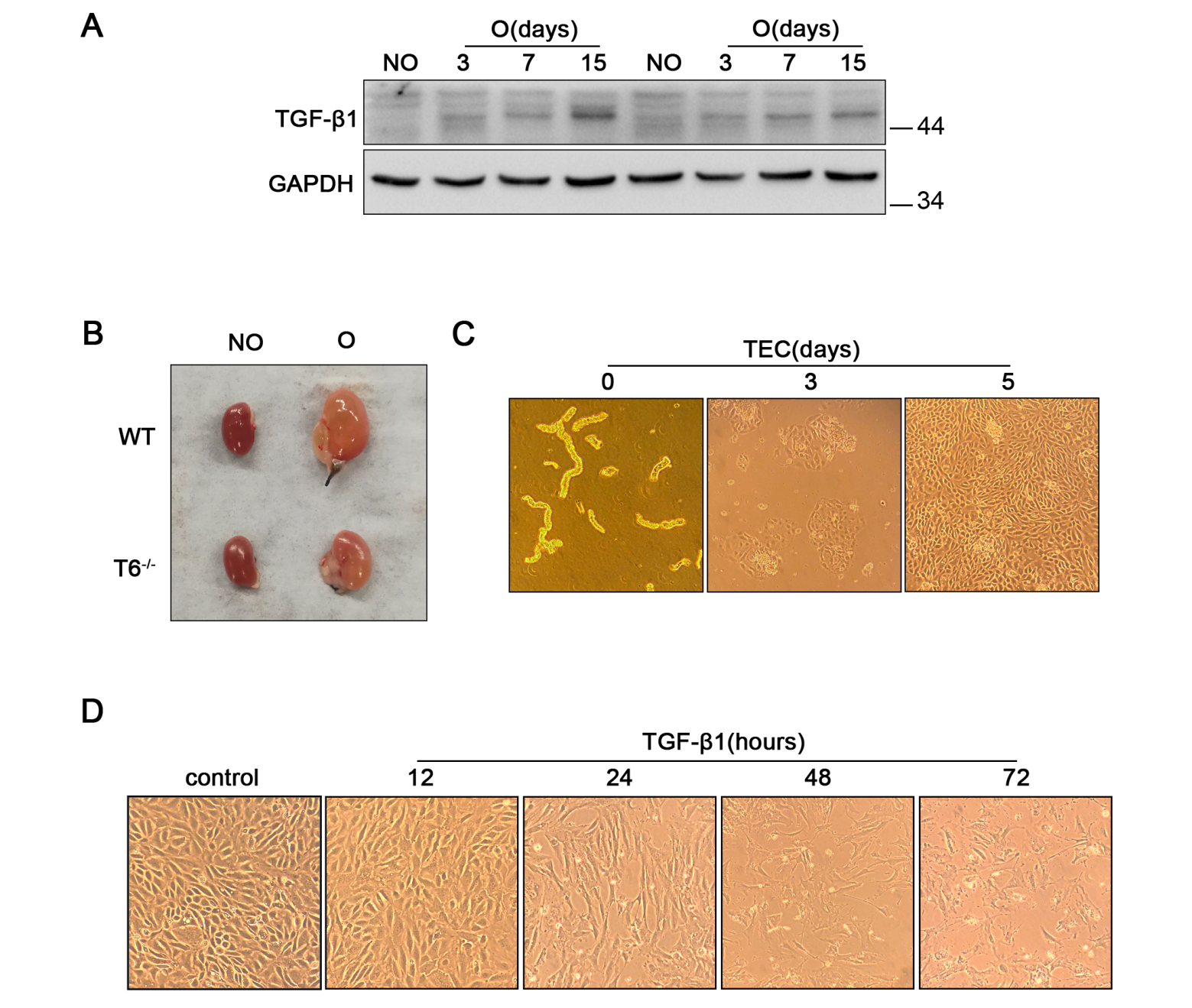


**Supplementary Figure S_2_** **The expression of TRPC3 and TRPC6 in TEC .** Expression level of TRPC6 (green) and TRPC3 (green) as well as nuclear stain DAPI (blue) in primary TEC with and without TGF-β1 stimulation for 72 h from WT **(A)** and TRPC6^-/-^ **(B)** mice detected by immunofluorescent staining. Scale bars, 20 µm.


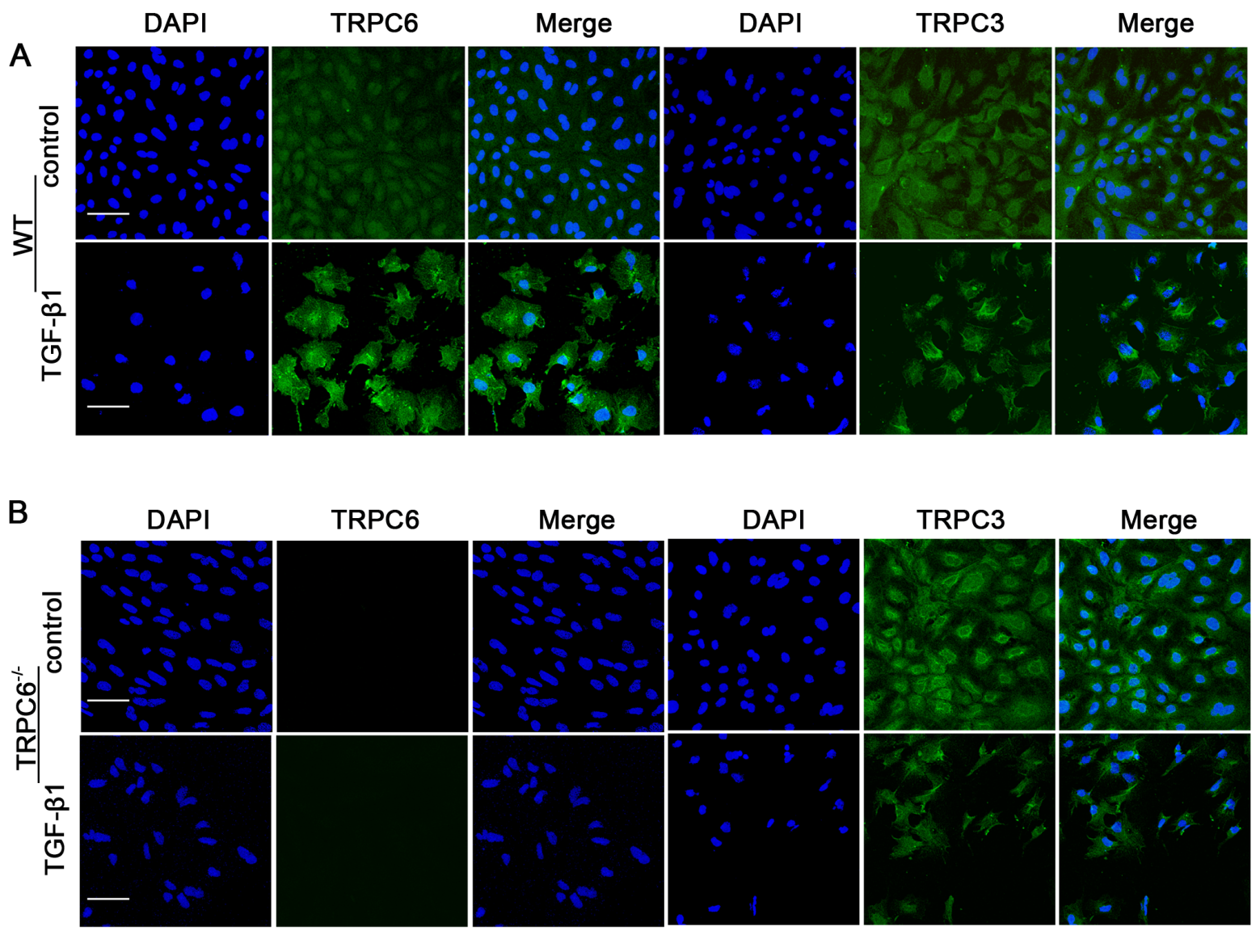


**Supplementary Figure S_3_** **The promotion of EMT and up-regulation of AKT/mTOR and ERK1/2 pathways in TEC of HYP9-stimulated group.** **(A)** Expression levels of EMT markers in primary TEC with and without HYP9 pretreatment detected by western blot. **(B)** Expression levels of the total and phosphorylated protein of AKT, mTOR, and ERK1/2 in primary TEC with and without HYP9 pretreatment detected by western blot.


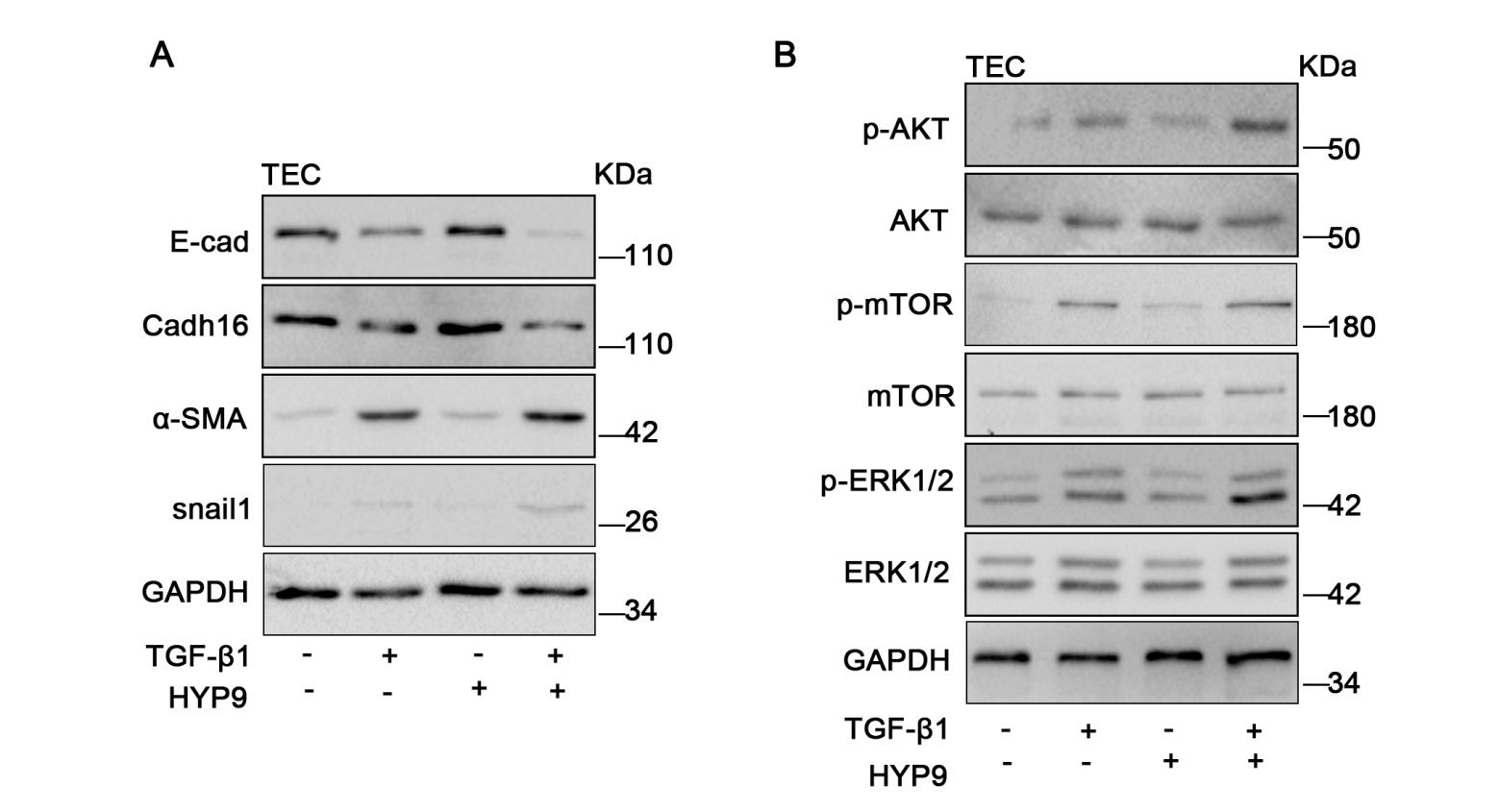

Supplement: Supplementary file 1 [file Table_1.DOCX]
